# Urinary metabolomics for discovering metabolic biomarkers of bladder cancer by UPLC-MS

**DOI:** 10.1186/s12885-022-09318-5

**Published:** 2022-02-28

**Authors:** Rui Wang, Huaixing Kang, Xu Zhang, Qing Nie, Hongling Wang, Chaojun Wang, Shujun Zhou

**Affiliations:** 1 Zibo Municipal Hospital, Zibo, Shandong 255400 China; 2 Department of clinical laboratory, Central Hospital of Xiangtan, Xiangtan, Hunan 411100 China; 3 Department of Urology, the First Affiliated Hospital, Zhejiang University School of Medicine, Hangzhou, Zhejiang 310003 China; 4 Yaneng Bioscience, Co., Ltd, Shenzhen, Guangdong 518100 China

**Keywords:** Bladder cancer, Urinary metabolomics, UPLC-MS, Potential biomarker, Diagnosis

## Abstract

Bladder cancer (BC) is one of the most frequent cancer in the world, and its incidence is rising worldwide, especially in developed countries. Urine metabolomics is a powerful approach to discover potential biomarkers for cancer diagnosis. In this study, we applied an ultra-performance liquid chromatography coupled to mass spectrometry (UPLC-MS) method to profile the metabolites in urine from 29 bladder cancer patients and 15 healthy controls. The differential metabolites were extracted and analyzed by univariate and multivariate analysis methods. Together, 19 metabolites were discovered as differently expressed biomarkers in the two groups, which mainly related to the pathways of phenylacetate metabolism, propanoate metabolism, fatty acid metabolism, pyruvate metabolism, arginine and proline metabolism, glycine and serine metabolism, and bile acid biosynthesis. In addition, a subset of 11 metabolites of those 19 ones were further filtered as potential biomarkers for BC diagnosis by using logistic regression model. The results revealed that the area under the curve (AUC) value, sensitivity and specificity of receiving operator characteristic (ROC) curve were 0.983, 95.3% and 100%, respectively, indicating an excellent discrimination power for BC patients from healthy controls. It was the first time to reveal the potential diagnostic markers of BC by metabolomics, and this will provide a new sight for exploring the biomarkers of the other disease in the future work.

**Supplementary Information:**

The online version contains supplementary material available at 10.1186/s12885-022-09318-5.

## Introduction

Bladder cancer (BC), also known as urinary bladder cancer, is the tenth most frequent cancer in the world (sixth in men and seventeenth in women), and its incidence is steadily rising worldwide, especially in developed countries, with approximately 550,000 new cases annually [1, 2]. Prolonged exposure to environmental and occupational chemicals could result in the tumorigenesis of BC. Among them, tobacco smoke is the main known cause, which is a possible explanation that greater tobacco smoke in men leads to the 4-fold gender discrepancy in BC incidence [1, 3, 4]. In addition, BC is a heterogeneous disease and possesses a high risk of morbidity and recurrence [5]. Among BC patients, it has primary and recurrent bladder cancer, and the stages of BC could be classified into T1, T2, T3, T4, Ta, *etc* [6]. The current BC diagnoses are mainly based on urinary cytology, cystoscopy and radiological imaging [6–8]. Cystoscopy is invasive, painful and costly, and it has low sensitivity for diagnosing high-grade superficial tumors. Particularly, it may lead to a high psychological burden for some patients once coupled with biopsy [7, 8]. Urinary cytology is a noninvasive test with high specificity, but poor sensitivity [9]. Therefore, it is urgent to seek more new noninvasive, sensitive and less expensive methods for BC diagnosis.

The reported biomarkers of bladder cancer mainly focused on the gene expression, such as CDK1, MAGEA3, *etc* [10]. Some of them lacked experimental validations. Meanwhile, gene markers might be failed since they could be regulated by the other proteins or signals. In recent years, metabolomics has proved to be a powerful technique for investigating the variation of endogenous small molecules during life activities in a high-throughput mode [10, 11]. Metabolites have played important roles in biological systems that diseases cause the disruption of biochemical pathways, and the metabolites changes observed in patients as primary indicators have been an important part of clinical practice [12]. Nowadays, metabolomics has been recognized as the preferred approach for biomarker identification, early disease diagnosis and searching related pathways [10, 13, 14]. For example, with the help of urine metabolomics, a marker discovery pipeline selected six putative markers from the metabolomic profiles, which could be employed for the discrimination of BC samples from hernia samples [15].

**Fig. 1:**
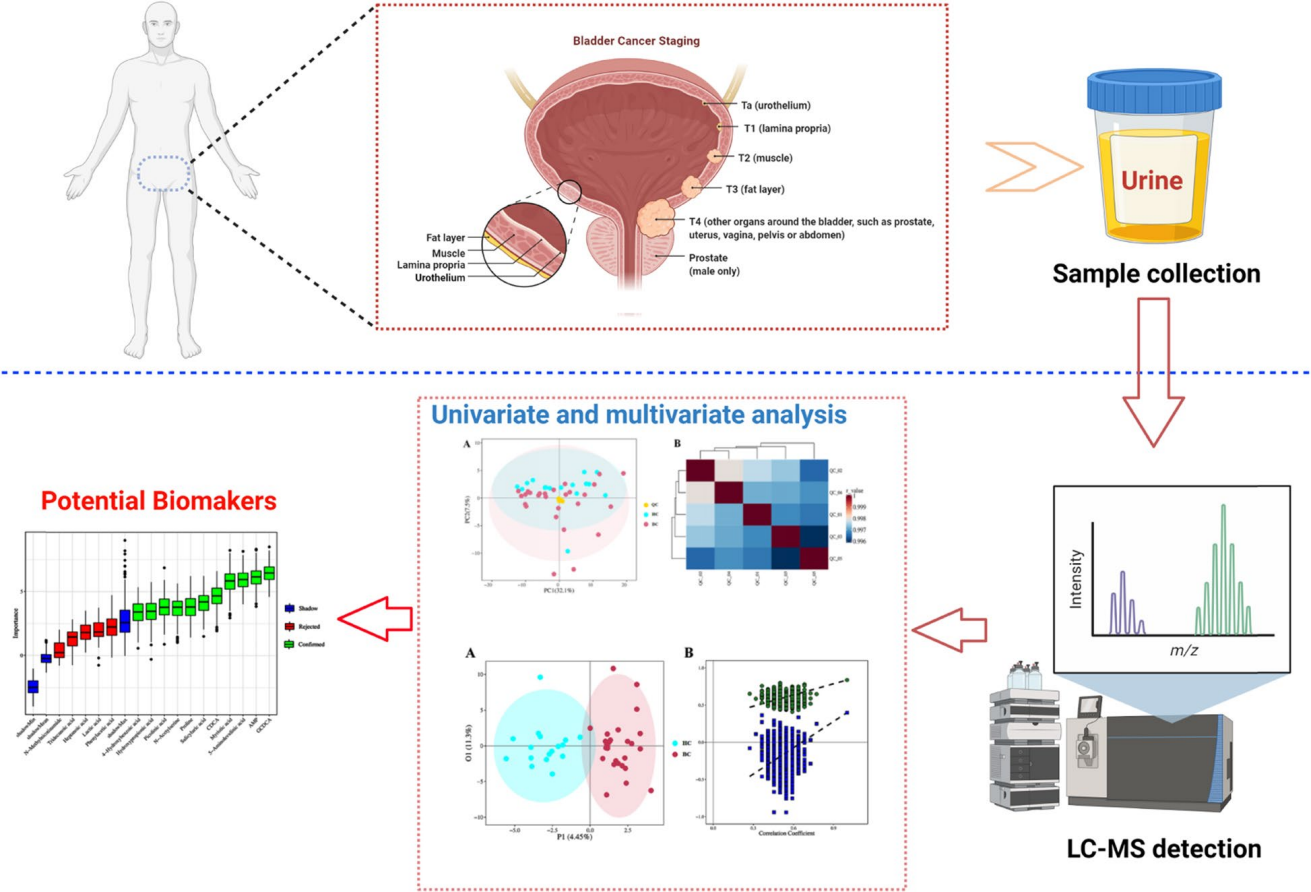
The workflow of urine biomarker discovery in bladder cancer

Mass spectrometry (MS) is a generally used platform for metabolomics analysis, and it is always coupled with advanced separation techniques such as gas chromatography (GC-MS), liquid chromatography (LC-MS) and/or others [16–18]. However, GC-MS is only suitable for analyzing volatile metabolites, resulting in the limited application. On the contrary, LC-MS has been widely used for metabolomics analysis benefitting from its high separation power and resolution [19, 20]. Therefore, in this study, a method by ultra-performance liquid chromatography coupled to mass spectrometry (UPLC-MS) was developed and applied to detect endogenous metabolites in urine from BC and healthy control groups. Multivariate statistical analysis methods were employed to identify significantly differential metabolites and potential biomarkers. The pattern recognition analytical techniques, including principal components analysis (PCA), partial least squares discriminant analysis (PLS-DA) and orthogonal partial least squares discriminant analysis (OPLS-DA), were used to comprehensively evaluate the metabolites that were present in any given biological case or that were connected to a specific disease condition (Fig. 1). As a result, a combinatorial biomarker panel, with high sensitivity and specificity, was explored and defined as core indicators in BC diagnosis.

## Materials and methods

### Chemicals and reagents

Formic acid was of analytical grade and obtained from Sigma-Aldrich (St. Louis, MO, USA). Methanol (Optima LC-MS grade), acetonitrile (Optima LC-MS grade), and isopropanol (Optima LC-MS grade) were purchased from Thermo Fisher Scientific (FairLawn, NJ, USA). Sable isotope-labeled internal standards and the derivatization regents, 3-nitrophenylhydrazine (3-NPH) and N-(3-(dimethylamino)propyl)-N′-ethylcarbodiimide (EDC)·HCl were purchased from Sigma-Aldrich (St. Louis, MO, USA). Ultra-pure water was produced by a Milli-Q system equipped with a LC-MS Pak filter (Millipore, Billerica, MA, USA).

All of the standards were purchased from TRC Chemicals (Toronto, ON, Canada), Sigma-Aldrich (St. Louis, MO, USA) and Steraloids Inc. (Newport, RI, USA). They were accurately weighed and dissolved in appropriate solutions to obtain individual stock solutions at the concentration of 5.0 mg mL^− 1^. Appropriate amount of each stock solution was mixed to get stock calibration solutions.

### Apparatus

An ultra-performance liquid chromatography coupled to tandem mass spectrometry (ACQUITY UPLC-Xevo TQ-S, Waters Corp., Milford, MA, USA) with an electrospray ionization (ESI) source was operated under positive and negative ion modes for the quantitation of metabolites. The UPLC-MS system was controlled by MassLynx 4.1 software. The chromatographic separations were carried out by an ACQUITY BEH C18 column (100 mm × 2.1 mm, 1.7 μm) (Waters, Milford, MA) at a flow rate of 0.4 mL min^− 1^. The mobile phases were consisted of 0.1% formic acid in water (solvent A) and acetonitrile/isopropanol (70:30, *v:v*) (solvent B), and a gradient elution program was set as follows: 0–1 min, 5% B; 1–5 min, 5–30% B; 5–9 min, 30–50% B; 9–12 min, 50–79% B; 12–15 min, 78–95% B; 15–16 min, 95–100% B; 16–18 min, 100% B. The main parameters of ESI source were optimized and adopted as follows: 1.2 kV (ESI^−^) and 3.2 kV (ESI^+^) of capillary voltage, 150 °C of source temperature, 550 °C of desolvation temperature, and 1200 L h^− 1^ of desolvation gas flow (N_2_). Collision-induced dissociation (CID) activation was used for the MS/MS fragmentation with an isolation width of *m/z* 3.0.

### Clinical samples

A total of 44 subjects, including 29 BC patients (BCs) and 15 healthy controls (HCs), were recruited at the First Affiliated Hospital, Zhejiang University School of Medicine. Among the collected BC patients, 19 were classified into high stage and 10 were low stage. The detailed information was showed in the supplementary materials Table S1. The experiment was approved by Zhejiang University Institutional Review Board, and informed consent forms were obtained from all participants. The diagnosis, staging and other information of BCs were obtained from the database for inpatients of the First Affiliated Hospital. The midstream urine was freshly collected in the morning after overnight fasting, then transferred into an Eppendorf tube, which was stored at − 80 °C before use.

### Urine sample preparation

Metabolomics analysis on urine samples was conducted by using the Q300 Metabolite Assay Kit (Human Metabolomics Institute, Inc., Shenzhen, Guangdong, China), referring to reported method with some modifications [21]. In brief, samples were firstly thawed on the ice-bath to reduce sample degradation. Then, 25 μL of urine was added to a 96-well plate, which was loaded to the Biomek 4000 workstation (Biomek 4000, Beckman Coulter, Brea, California, USA) [21]. The cold methanol containing partial internal standards was automatically added to each sample, and the samples were subsequently vortexed for 5 min [22]. After centrifugation for 30 min at 4000×*g* (Allegra X-15R, Beckman Coulter, Indianapolis, IN, USA), 30 μL of supernatant and 20 μL of fresh derivative reagents (200 mM 3-NPH in 75% methanol and 96 mM EDC-6% pyridine solution in methanol) were added to each well of a new clean 96-well plate [22]. After derivatization at 30 °C for 60 min, each sample was diluted by 350 μL of cold 50% methanol and stored at − 20 °C for 20 min. After centrifugation with the conditions of 4000×*g* and 4 °C for 30 min, 135 μL of supernatant and 15 μL of internal standards were added to each well on a new 96-well plate. And the remaining wells were filled with serial diluted derivatized stock standards. At last, the sample plate was sealed for the subsequent UPLC-MS analysis.

### Quality control approach for metabolomic analysis

Periodic analysis of real samples together with quality control (QC) samples was applied in this study to ensure the excellent quality of metabolic profiling [12]. In detail, five injections of QC samples were put in the analytical platform in the first instance. Next, before inserting 5 samples, one QC sample was breathed into the sample set in order. The QC samples were prepared by a mixture of BCs and HCs samples with the same volumes.

### Data analysis and statistical analysis

The raw MS data files were processed by Targeted Metabolome Batch Quantification (TMBQ) software (v1.0, Human Metabolomics Institute, Inc., Shenzhen, Guangdong, China) to perform peak integration, calibration, and quantitation for each metabolite [23]. Identified metabolites were further annotated using the Kyoto Encyclopedia of Genes and Genomes (KEGG) database (http://www.genome.jp/kegg/) and Human Metabolome Database (HMDB) (http://www.hmdb.ca/) [24]. Metaboanalyst (https://www.metaboanalyst.ca/) was employed to perform the metabolic pathway enrichment of differential metabolites. PCA and OPLS-DA were carried out by metaX software [25]. Univariate analysis (t-test) was employed to calculate the statistical significance (*P*-value) [26]. The metabolites with variable importance in the projection (VIP) > 1, *P*-value < 0.05 and |fold change (FC)| >0 were regarded as differential metabolites [27].

## Results

### Clinical information of participants

An untargeted metabolomics method was used to study urine samples from 29 BCs and 15 HCs. The participants’ clinical information is summarized in Table 1. These participants aged from 48 to 92 years old with an average age of 68.2. In the BCs, 21 (72.4%) were male and 8 (27.6%) were female. In the HCs, 12 (80.0%) were male and 3 (20.0%) were female.

**Table 1:** Characteristics of enrolled patients

Groups	No. of subjects	Gender	
		Male	Female
**Sample number**	44	33	11
BC patient	29	21	8
HC control	15	12	3
**Among patients**			
Age range	68.2 (48–92)		
**Stage**			
Ta	11	7	4
T1	6	6	0
T2	5	2	3
T3	4	4	0
T4	3	2	1
MIBC	12	8	4
NMIBC	17	13	4
High grade	19	12	7
Low grade	10	9	1
Primary	18	14	4
Recurrence	11	7	4

### QC sample analysis

**Fig. 2:**
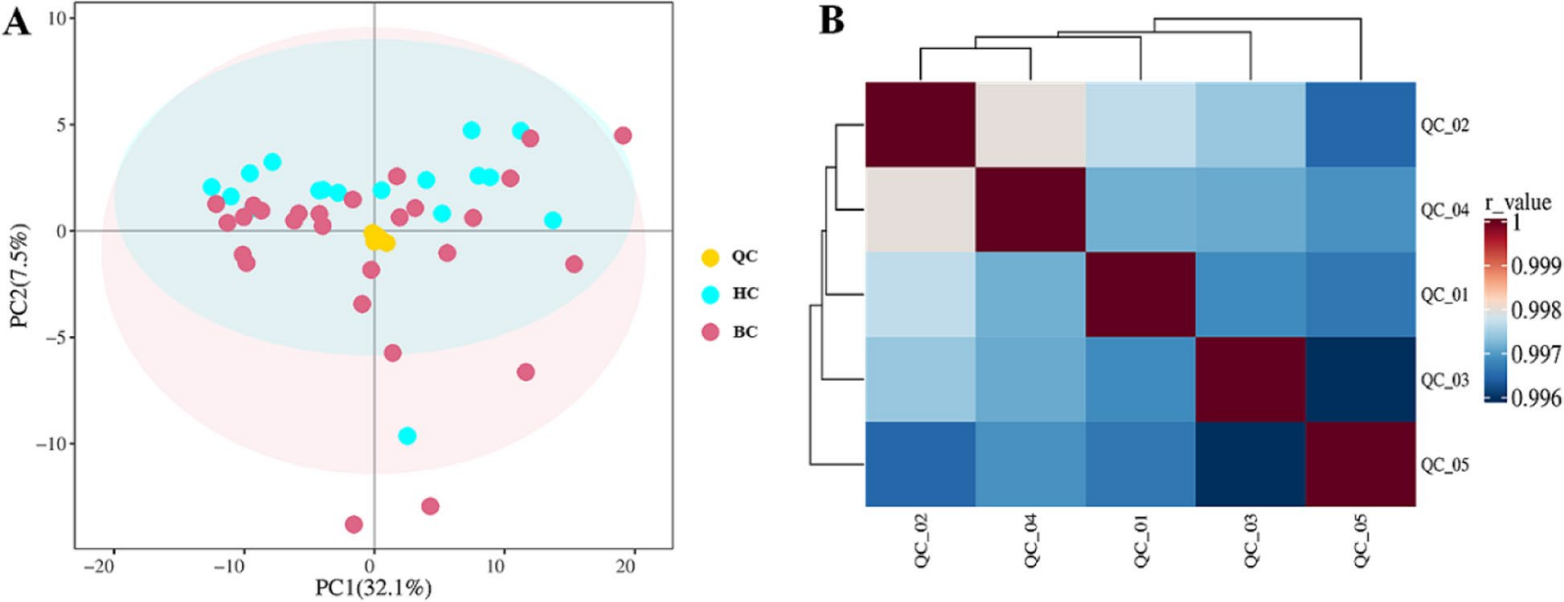
**A** PCA score plot for QC samples and tested samples. Yellow dots denote QC samples, blue dots are HC samples and red dots represent BC samples. **B** correlation heat map for QC samples

PCA analysis was performed based on QC samples and tested samples. The PCA score plot is shown in Fig. 2A. The result indicated that QC samples formed a cluster without any obvious drift during metabonomic profiling. In addition, the pearson correlation (calculated by Pearson Correlation Coefficient) of any two QC samples was within 0.996 and 1 (Fig. 2B). These results demonstrated the current metabolomics data had good stability and reproducibility.

### Urine metabolomics data analysis

**Fig. 3:**
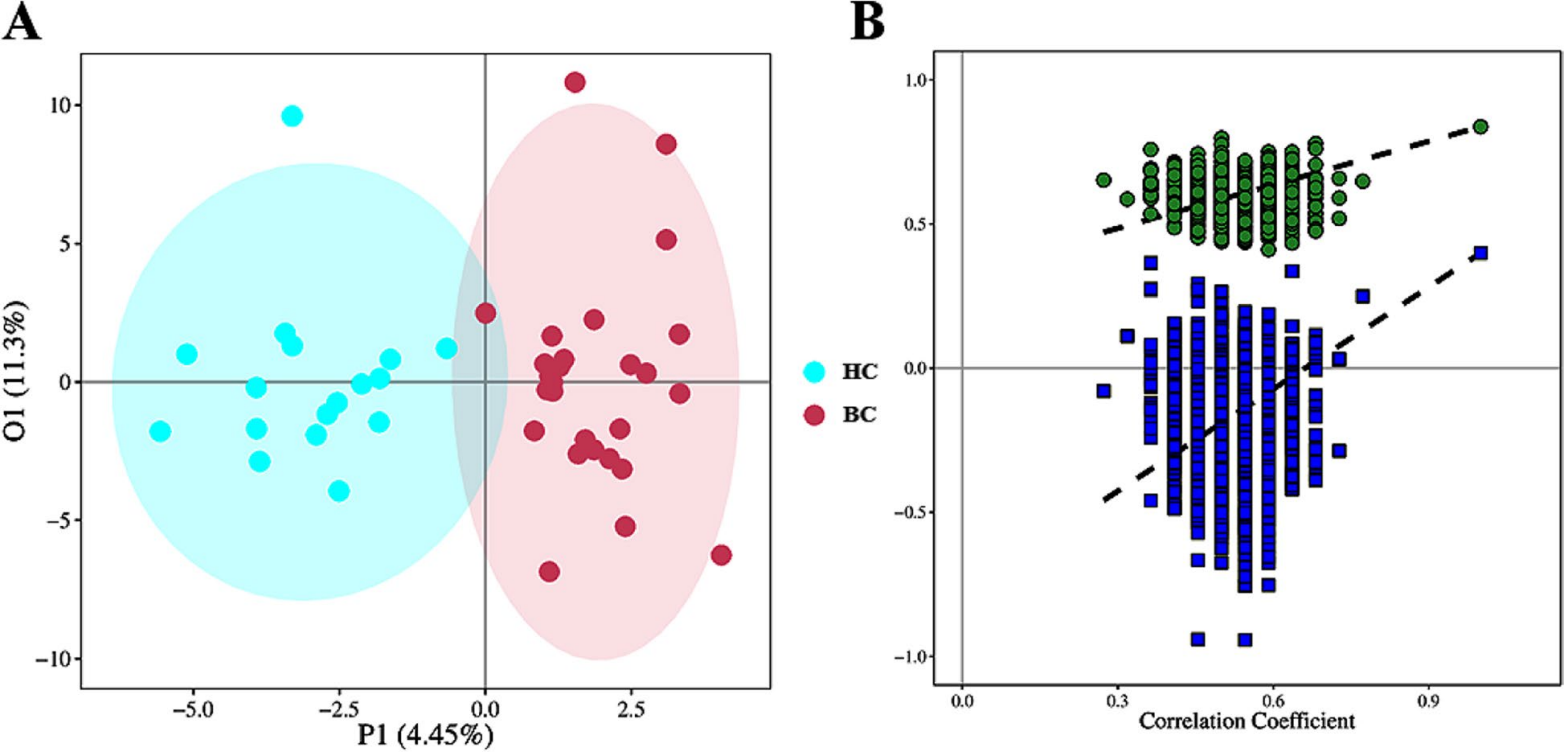
**A** OPLS-DA score plot for HC and BC groups. Blue circles and red circles represent data for HC and BC samples, respectively. (B) The correlation coefficient (R^2^) distribution plot of the permutation test for the OPLS-DA model

Based on the untargeted metabolomics technique, a total of 208 metabolites were identified in the urine samples. To evaluate the discriminating power of the obtained 208 metabolites, we performed OPLS-DA analysis for the urine samples from 29 BCs and 15 HCs (Fig. 3A). The OPLS-DA model was constructed by performing 7-fold cross-validation, and the result showed satisfactory modeling and prediction with 1 predictive component and 2 orthogonal components (R^2^X_cum_ = 0.157, R^2^Y_cum_ = 0.837, Q^2^_cum_ = 0.399). To avoid model over-fitting, the model was further validated with a permutation multivariate analysis of variance (PERMANOVA), and the result indicated that the probability of this model randomly occurring was less than 0.001 (Fig. 3B). From these satisfactory results, the metabolic profiling of BC patients showed significantly discriminative potential from that of HCs.

### Identification of metabolic biomarkers

**Fig. 4:**
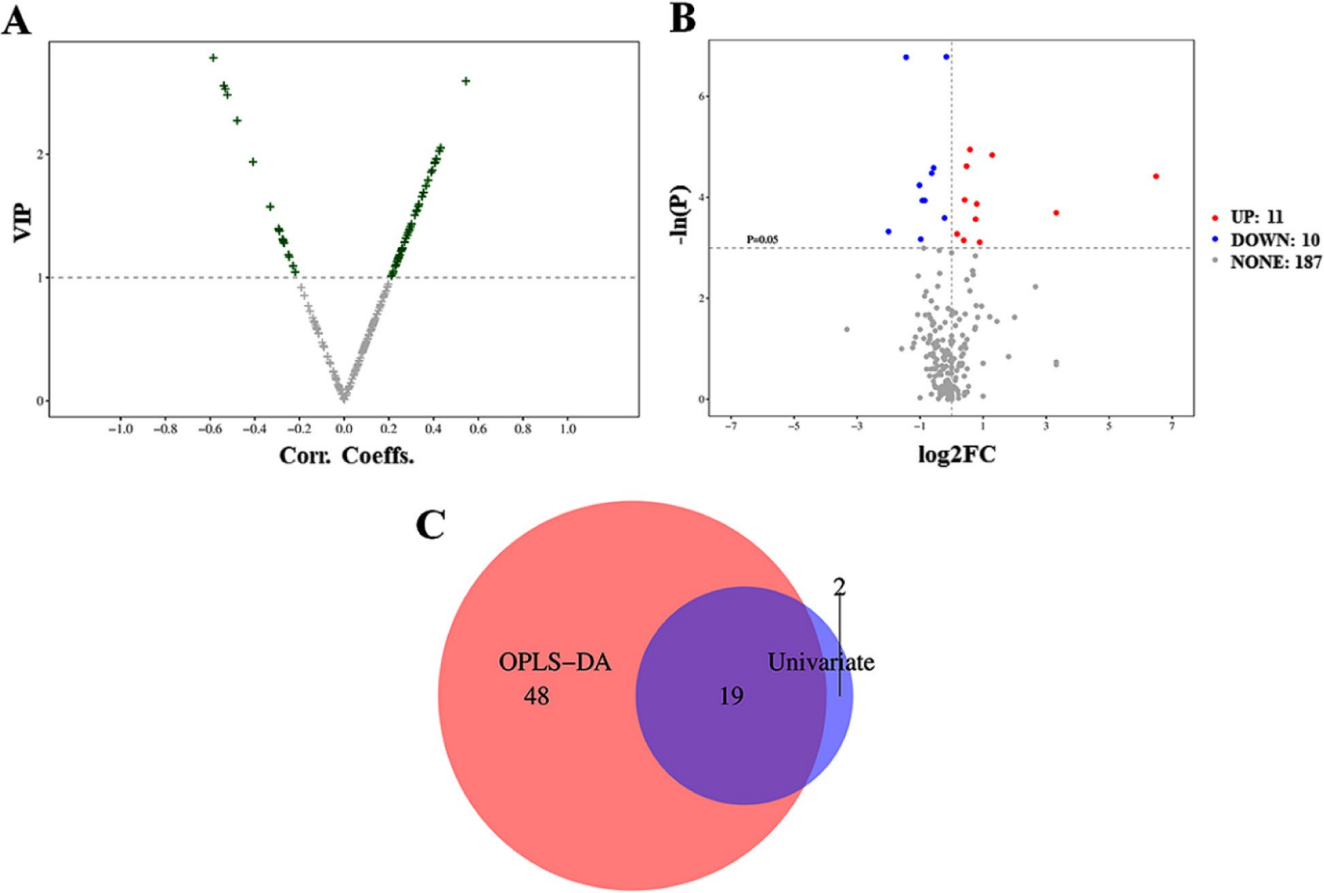
**A** Volcano plot of VIP scores from OPLS-DA model. The green crosses represent the metabolites with VIP>1 and the grey crosses represent the metabolites with VIP ≤ 1. **B** Volcano plot with the univariate statistical test (−ln P) and the magnitude of the change (log2FC) of metabolites. Red points represent the metabolites with *P*-value < 0.05 and FC>0. Blue points represent the metabolites with *P*-value < 0.05 and FC < 0. Grey points represent the metabolites with *P*-value>0.05. **C** Venn diagram integrating results from volcano-plots of OPLS-DA model and univariate statistical test

In the current study, we applied two types of analysis to identify the significantly changed metabolites in BC patients and explore potential biomarkers for diagnosis of BC. Firstly, VIP scores of obtained 208 metabolites were extracted from the OPLS-DA model. The volcano plot of VIP scores for these metabolites is shown in Fig. 4A. The green crosses in the volcano plot indicated 67 significantly changed metabolites with VIP > 1. Secondly, t-test was employed to calculate the *P*-value and fold change (FC), which was shown in the volcano plot (Fig. 4B). As a result, 21 metabolites with *P* value < 0.05 and |FC|>0 were highlighted as differential metabolites, including 11 upregulation (marked in red) and 10 downregulation (marked in blue). In consideration of stage and gender influence, we carefully analyzed the data (Fig. S1 and Fig. S2). The top 10 metabolites were listed in Table S2 and S3. There were common metabolites, such as AMP, GUDCA, etc. Meanwhile, different metabolites between low grade and high grade were also found, such as N-Methylnicotinamide. There was some difference based on stage or gender analysis. However, due to the limitation of samples, more samples should be collected to make a direction. All the metabolites were presented in the top 21 metabolites of changed metabolites in BC patients.

**Fig. 5:**
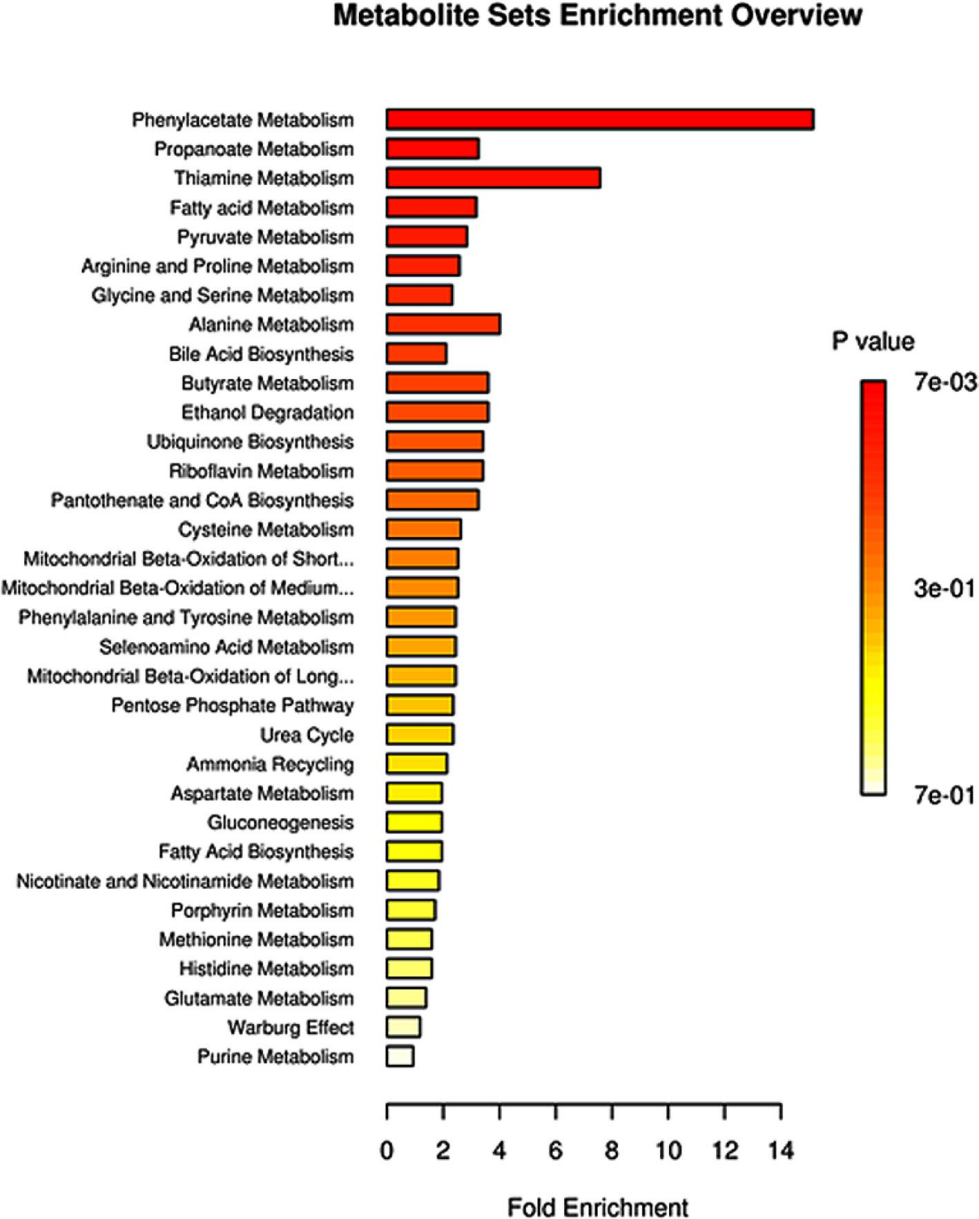
Differential metabolite pathway analysis. The color depth and column length indicate the disturbance degree of the pathway

Finally, a Venn diagram was plotted to integrate the selected results from the OPLS-DA model and univariate analysis (Fig. 4C). In the overlapped area of the Venn diagram, a total of 19 metabolites could meet the following criteria of VIP > 1 and *P*-value < 0.05 and |FC|>0, which were regarded as metabolic biomarkers. Their detailed information is listed in Table 2. Based on the relative abundance of differential metabolites, pathway enrichment results showed that 33 metabolic pathways were identified in Small Molecule Pathway Database (SMPDB). Among them, 7 pathways, including phenylacetate metabolism, propanoate metabolism, fatty acid metabolism, pyruvate metabolism, arginine and proline metabolism, glycine and serine metabolism, and bile acid biosynthesis, were significantly enriched with at least 2 annotated metabolites (Fig. 5). The detailed pathway enrichment results are displayed in Table 3.

**Table 2:** 19 differential metabolites annotated in KEGG or HMDB database

Metabolite	Class	HMDB	KEGG	***P***-value	FC	VIP
Hydroxypropionic acid	Organic Acids	HMDB0000700	C01013	0.0195	0.5273	1.2951
AMP	Nucleotides	HMDB0000045	C00020	0.0079	2.4444	1.1625
Lactic acid	Organic Acids	HMDB0000190	C00186	0.0446	1.8547	1.6818
Picolinic acid	Pyridines	HMDB0002243	C10164	0.0102	0.6731	1.2883
4-Hydroxybenzoic acid	Benzoic Acids	HMDB0000500	C00156	0.0114	0.6455	1.3049
Phenylacetic acid	Benzenoids	HMDB0000209	C07086	0.0429	1.3069	1.9231
Salicyluric acid	Benzoic Acids	HMDB0000840	C07588	0.0144	0.4935	1.378
Proline	Amino Acids	HMDB0000162	C00148	0.0209	1.7364	1.0882
N-Acetylserine	Amino Acids	HMDB0002931	NA	0.042	0.5078	1.0862
5-Aminolevulinic acid	Amino Acids	HMDB0001149	C00430	0.0011	0.3679	2.5489
N-Methylnicotinamide	Pyridines	HMDB0003152	NA	0.0283	1.6952	1.783
Heptanoic acid	Fatty Acids	HMDB0000666	C17714	0.0378	1.129	2.0465
GUDCA	Bile Acids	HMDB0000708	NA	0.0121	90.0	1.9545
CDCA	Bile Acids	HMDB0000518	C02528	0.0099	1.3894	2.5883
GCDCA	Bile Acids	HMDB0000637	C05466	0.0071	1.5	1.4274
Tridecanoic acid	Fatty Acids	HMDB0000910	C17076	0.0276	0.8571	2.2681
Myristic acid	Fatty Acids	HMDB0000806	C06424	0.0011	0.8913	2.5243
3-Hydroxylisovalerylcarnitine	Carnitines	NA	NA	0.0195	0.5544	1.3705
Palmitoylcarnitine	Carnitines	HMDB0000222	C02990	0.0249	10.0	1.3128

**Table 3:** SMPDB pathway enrichment

Pathway	Total	Expected	Hits	***P***-value	Adjust ***P***-value	FDR	Enriched compounds
Phenylacetate Metabolism	9	0.132	2	0.0068	0.666	0.666	AMP; Phenylacetic acid
Propanoate Metabolism	42	0.615	2	0.123	1	1	AMP; Hydroxypropionic acid
Fatty acid Metabolism	43	0.63	2	0.128	1	1	AMP; Palmitoylcarnitine
Pyruvate Metabolism	48	0.703	2	0.153	1	1	AMP; Lactic acid
Arginine and Proline Metabolism	53	0.776	2	0.18	1	1	AMP; Proline
Glycine and Serine Metabolism	59	0.864	2	0.212	1	1	AMP; 5-Aminolevulinic acid
Bile Acid Biosynthesis	65	0.952	2	0.246	1	1	CDCA; GCDCA
Thiamine Metabolism	9	0.132	1	0.125	1	1	AMP
Alanine Metabolism	17	0.249	1	0.223	1	1	AMP
Butyrate Metabolism	19	0.278	1	0.246	1	1	AMP
Ethanol Degradation	19	0.278	1	0.246	1	1	AMP
Ubiquinone Biosynthesis	20	0.293	1	0.258	1	1	4-Hydroxybenzoic acid
Riboflavin Metabolism	20	0.293	1	0.258	1	1	AMP
Pantothenate and CoA Biosynthesis	21	0.308	1	0.269	1	1	AMP
Cysteine Metabolism	26	0.381	1	0.322	1	1	AMP
Mitochondrial Beta-Oxidation of Short Chain Saturated Fatty Acids	27	0.396	1	0.332	1	1	AMP
Mitochondrial Beta-Oxidation of Medium Chain Saturated Fatty Acids	27	0.396	1	0.332	1	1	AMP
Phenylalanine and Tyrosine Metabolism	28	0.41	1	0.342	1	1	AMP
Selenoamino Acid Metabolism	28	0.41	1	0.342	1	1	AMP
Mitochondrial Beta-Oxidation of Long Chain Saturated Fatty Acids	28	0.41	1	0.342	1	1	AMP
Pentose Phosphate Pathway	29	0.425	1	0.352	1	1	AMP
Urea Cycle	29	0.425	1	0.352	1	1	AMP
Ammonia Recycling	32	0.469	1	0.381	1	1	AMP
Aspartate Metabolism	35	0.513	1	0.409	1	1	AMP
Gluconeogenesis	35	0.513	1	0.409	1	1	Lactic acid
Fatty Acid Biosynthesis	35	0.513	1	0.409	1	1	Myristic acid
Nicotinate and Nicotinamide Metabolism	37	0.542	1	0.426	1	1	AMP
Porphyrin Metabolism	40	0.586	1	0.452	1	1	5-Aminolevulinic acid
Methionine Metabolism	43	0.63	1	0.477	1	1	AMP
Histidine Metabolism	43	0.63	1	0.477	1	1	AMP
Glutamate Metabolism	49	0.718	1	0.523	1	1	AMP
Warburg Effect	58	0.85	1	0.586	1	1	Lactic acid
Purine Metabolism	74	1.08	1	0.678	1	1	AMP

### Potential biomarkers for BC diagnosis

**Fig. 6:**
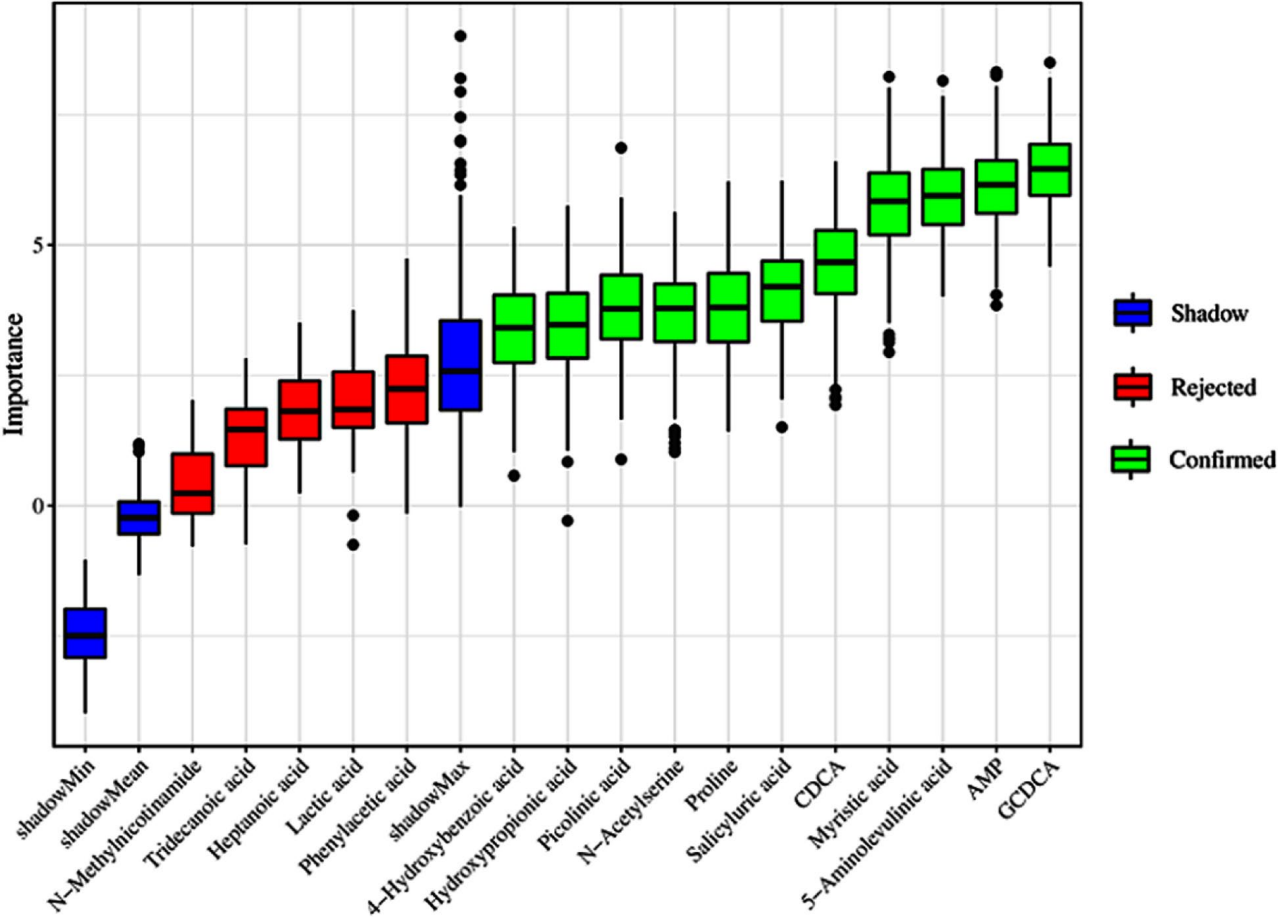
Relative importance (RI) of metabolites calculated by boruta algorithm

**Fig. 7:**
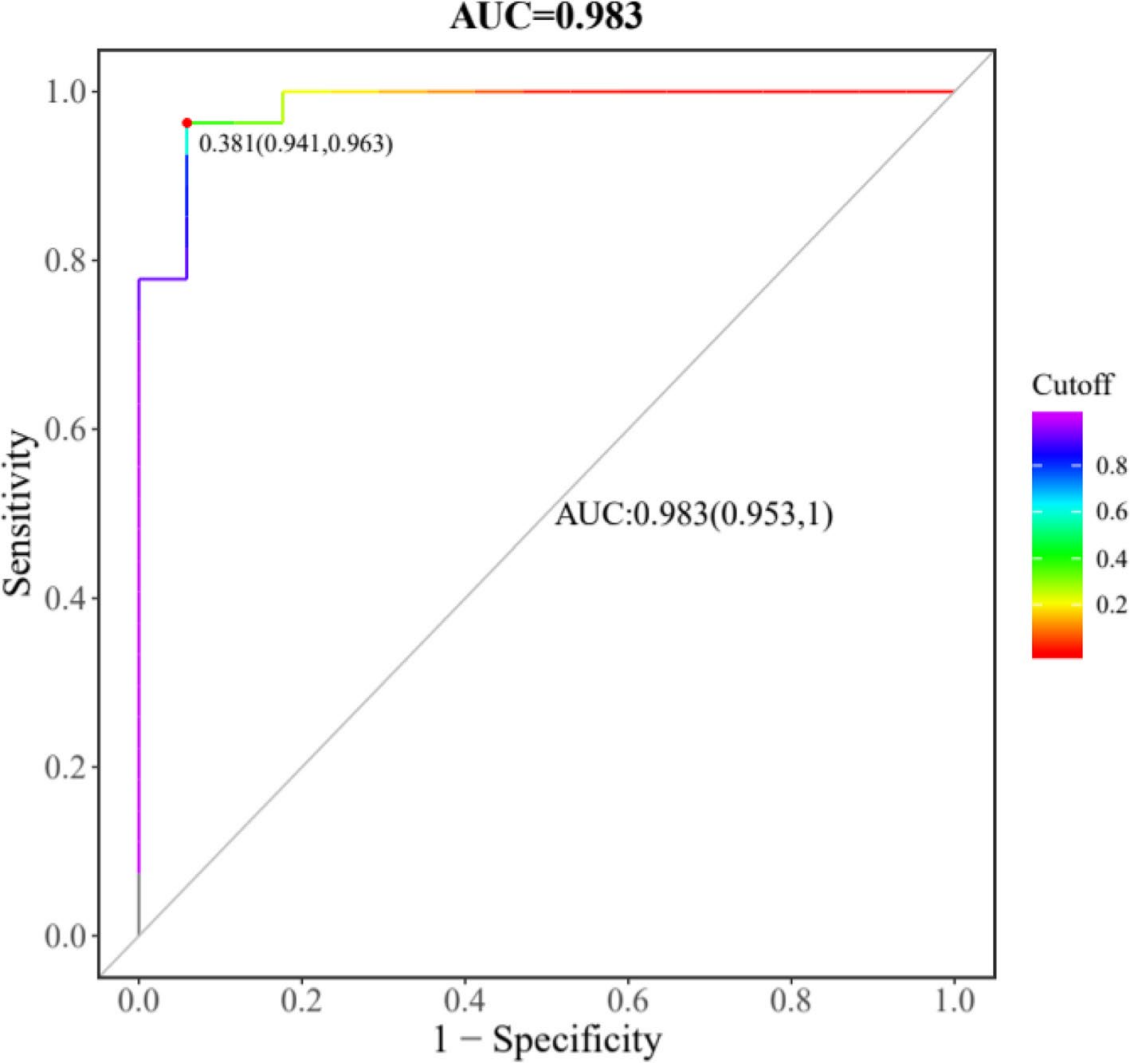
Receiver operating characteristic (ROC) curve of a logistic regression model for distinguishing BCs from HCs by using 11 potential biomarkers conjunctively

In order to find out candidate biomarkers from 19 identified differential metabolites, we carried out random forest (RF), support vector machine (SVM) and boruta analysis in sequence. First, we got union set between top 10 metabolites from RF and top 10 metabolites from SVM, which were employed to carry out further selection of potential biomarkers using boruta analysis. In this study, the result of the boruta algorithm for selecting the most important metabolites is shown in Fig. 6, and a total of 11 metabolites, namely glycochenodeoxycholic acid (GCDCA), adenosine monophosphate (AMP), 5-Aminolevulinic acid, myristic acid, chenodeoxycholic acid (CDCA), salicyluric acid, proline, N-Acetylserine, picolinic acid, hydroxypropionic acid and 4-Hydroxybenzoic acid, were labeled as “Confirmed”, which could be used for model building and prediction. These 11 selected potential biomarkers were further combined by logistic regression (LR) model to build the biomarker panel, and the final receiving operator characteristic (ROC) curve is shown in Fig. 7. It can be observed that the biomarker panel had an area under the curve (AUC) of 0.983 and the values of sensitivity and specificity reached 95.3% and 100% at the best cut-off points.

## Discussion

Bladder cancer is one of the most prevalent malignancies of the urinary system, which is related to multiple factors, such as genetic susceptibility, environmental exposure as well as unhealthy lifestyles [5]. In recent years, there is a trend of increased incidence and mortality worldwide for BC [28, 29]. As is known, metabolomics has proved to be a powerful technique for investigating the variation of endogenous small molecules during life activities in a high-throughput mode [10], which could help us to identify potential metabolic biomarkers and study possible mechanisms for BC. Because bladder is a temporary storage of urine and the urinary metabolic characteristics could closely reveal changes that occur during pathological conditions, urinary metabolomics has some advantages over other biological fluids, such as noninvasive collection and convenient storage. In the present study, a nontargeted UPLC-MS method was applied to profile the metabolites of urine from 29 BC patients and 15 healthy subjects. A group of 19 metabolites was discovered as differently expressed biomarkers, which mainly related to phenylacetate metabolism, propanoate metabolism, fatty acid metabolism, pyruvate metabolism, etc. (Table 3). In addition, the combination of 11 potential biomarkers showed an excellent discrimination capability of BCs from HCs, which could be potentially used for clinical diagnosis, prognosis monitoring, and early detection of BC patients.

### Significantly altered metabolites and related pathways

Among the 19 differential metabolites, the changes of AMP, glycoursodeoxycholic acid (GUDCA) and palmitoylcarnitine were the most dramatic ones with FC>2. Adenosine monophosphate, known as AMP, is a nucleotide that is found in RNA. AMP can be produced during adenosine triphosphate (ATP) synthesis by the enzyme adenylate kinase urine (Human Metabolome Database). The AMP-activated protein kinase (AMPK) is sensitive to cellular AMP/ATP ratio, in which a high AMP or low ATP level activates AMPK [30, 31]. In the present research, the content of AMP in BCs was higher than that in HCs. AMPK activation could provide a growth advantage to tumor cells by regulating cellular metabolic plasticity, thus providing tumor cells the flexibility to adapt to metabolic stress [32]. The role of core enzyme (AMPK) was analyzed by using Onconmine and cBIO portal. As showed in the following Fig. S3, it was found that the survival probability of Protein Kinase AMP-Activated Catalytic Subunit Alpha 2 (PRKAA2, one sub-type of AMPK) altered patients was relatively lower than those without alteration. Higher expression of AMP could result in the worse survival conditions.

The potential biomarkers of GUDCA, CDCA and GCDCA were also belonging to the group of bile acids, and they were all up-regulated in urine from BC patients as compared to the HC group. Bile acids are physiological detergent molecules, so are highly cytotoxic. Meanwhile, it is found that conjugated bile acids can activate the sphingosine 1-phosphate receptor 2 that activates intracellular ERK1/2 and AKT signaling to promote the invasive growth of cholangiocarcinoma, which is commonly associated with chronic cholestasis [33], and overexpression of bile acids in urine might result in worse condition of urinary bladder, even cancer. It was reported that bile acids were implicated as etiologic agents in cancer of the gastrointestinal tract, including cancers of the esophagus, stomach, small intestine, liver, biliary tract, pancreas and colon [34]. The bile acids could generate cellular reactive oxygen species and induce multiple stresses on cells including DNA damage, endoplasmic reticulum stress and mitochondrial damage, which lead to genomic instability, apoptosis, necrosis, autophagic cell death, etc. [35–38].

Palmitoylcarnitine is a well-known intermediate in mitochondrial fatty acid oxidation, which naturally exists in blood, feces, saliva and urine. In this study, the content of palmitoylcarnitine on bladder cancer patients was 10 times higher than that in healthy subjects. Some studies have reported the accumulation of palmitoy lcarnitine in diabetes mellitus type II, obesity and kidney cancer [39–41]. It was found that the increased contents of most acylcarnitines in the urine of cancer patients together with high cancer grades in those patients, and higher chain length acylcarnitines, such as palmitoylcarnitine, showed inhibitory effects on nuclear factor kappa-B (NF-kB) activation, indicating an immune modulatory effect [41]. Besides, it was reported that palmitoylcarnitine might represent a potential biomarker of the metabolic dysfunction associated with prostate cancer [42]. The result showed that, at physiological levels of palmitoylcarnitine, there were no effects on the prostate cancer cells; however, at high levels of palmitoylcarnitine, it drove tumor development through inducing key inflammatory cytokines and gene expression associated with glycolysis.

Interestingly, salicylic acid was a characteristic index presented in the urine samples of BC. Through analyzing the drug administration, we found that some of BC patients often used salicylic acid-related drugs, such as aspirin. This indicated that overdose of salicylic acid-related drugs might contribute to the tumorigenesis of BC. Due to the small samples in our work, more subjects were needed to be collected to validate this speculation.

### Diagnosis of BCs from HCs

The AUC was 0.983 for BC diagnosis, with 95.3% sensitivity and 100% specificity, showing satisfactory discrimination power by the eleven-biomarker panel. The results suggested that the eleven-biomarker panel could be used for the diagnosis of BCs from HCs. In addition, besides N-Acetylserine, all other potential biomarkers could individually achieve a high AUC value higher than 0.7 with satisfactory sensitivity and specificity. It should be noted that, because these 11 differential metabolites were chosen based on boruta algorithm using 29 BCs and 15 HCs, this predictive accuracy may be biased upward. Thus, the additional verification set should include a wider range of subjects, and their ages and gender should be carefully matched in the further study.

## Conclusion

In this study, we established and applied a UPLC-MS based metabolomics to investigate the metabolite difference in urine from 29 bladder cancer patients and 15 healthy subjects. An obvious discrimination was obtained by OPLS-DA analysis based on the identified metabolites between bladder cancer patients and healthy controls. In addition, 19 metabolites were discovered as differently expressed biomarkers in the two groups. Based on boruta analysis, 11 of them were selected and combined to test the potentiality of diagnosis of BC by using LR model. The AUC, sensitivity and specificity of ROC curve were 0.983, 95.3% and 100%, respectively, showing an excellent discrimination power for BC diagnosis.

## Supplementary Information

**Additional file 1.**

## Data Availability

The datasets used and/or analysed during the current study are available from the corresponding author on reasonable request.
