# Supplementary material for: Urinary metabolomics for discovering metabolic biomarkers of bladder cancer by UPLC-MS
Source: BMC Cancer. 2022 Feb 28;22:214. doi: 10.1186/s12885-022-09318-5 (PMC8883652; doi:10.1186/s12885-022-09318-5)
Supplement: Supplementary file 1 — Additional file 1. [file 12885_2022_9318_MOESM1_ESM.docx]

**Table S1.** The degree information of 29 BC patients.

| **No.** | **Gender** | **Age** | **Stage** | **Degree** |
| --- | --- | --- | --- | --- |
| 1 | Male | 79 | T4aN2M0 | High |
| 2 | Female | 51 | T2 | High |
| 3 | Male | 56 | T1 | High |
| 4 | Male | 64 | Ta | Low |
| 5 | Male | 48 | Ta | Low |
| 6 | Male | 65 | T1 | High |
| 7 | Male | 63 | T2 | High |
| 23 | Male | 70 | Ta | Low |
| 24 | Female | 78 | T4N1M0 | High |
| 25 | Male | 69 | Ta | Low |
| 26 | Male | 68 | T4 | High |
| 27 | Male | 62 | T3 | High |
| 28 | Male | 53 | T3 | High |
| 29 | Male | 65 | Ta | Low |
| 30 | Male | 62 | Ta | Low |
| 31 | Male | 68 | T1 | High |
| 32 | Female | 81 | T2 | High |
| 33 | Male | 72 | T1 | High |
| 34 | Male | 92 | T1 | High |
| 35 | Male | 59 | Ta | Low |
| 36 | Male | 73 | T3 | High |
| 37 | Female | 66 | Ta | Low |
| 38 | Male | 53 | T1 | Low |
| 39 | Female | 81 | Ta | High |
| 40 | Male | 82 | T2b | Low |
| 41 | Female | 57 | T2bN1M0 | High |
| 42 | Female | 87 | Ta | High |
| 43 | Female | 70 | Ta | High |
| 44 | Male | 85 | T3 | High |

**Table S2.** The top 10 characteristic metabolites in high and low BC patients.

| **No** | **High grade** | **Low grade** |
| --- | --- | --- |
| 1 | Heptanoic acid | Myristic acid |
| 2 | AMP | Citrulline |
| 3 | Propionic acid | Linoleic acid |
| 4 | Proline | GUDCA |
| 5 | Malic acid | N-Methylnicotinamide |
| 6 | Picolinic acid | Palmitoleic acid |
| 7 | 5-Aminolevulinic acid | AMP |
| 8 | GUDCA | 7-DHCA |
| 9 | Fumaric acid | GCDCA |
| 10 | 3-Hydroxylisovalerylcarnitine | 2-Furoic acid |

**Table S3.** The top 10 characteristic metabolites in male and female BC patients.

| No. | Male | Female |
| --- | --- | --- |
| 1 | Myristic acid | Tridecanoic acid |
| 2 | Propionic acid | Salicyluric acid |
| 3 | 5-Aminolevulinic acid | Shikimic acid |
| 4 | Malic acid | Hexanylcarnitine |
| 5 | 2-Furoic acid | GDCA |
| 6 | 4-Hydroxybenzoic acid | Octanoylcarnitine |
| 7 | Heptanoic acid | Dodecanoylcarnitine |
| 8 | AMP | Picolinic acid |
| 9 | Acetylglycine | LCA |
| 10 | GUDCA | Decanoylcarnitine |

**Figure S1.** The OPLS-DA score plot for HC and high and low grade BC patients.

**Figure S2.** The OPLS-DA score plot for HC and BC groups in male and female.


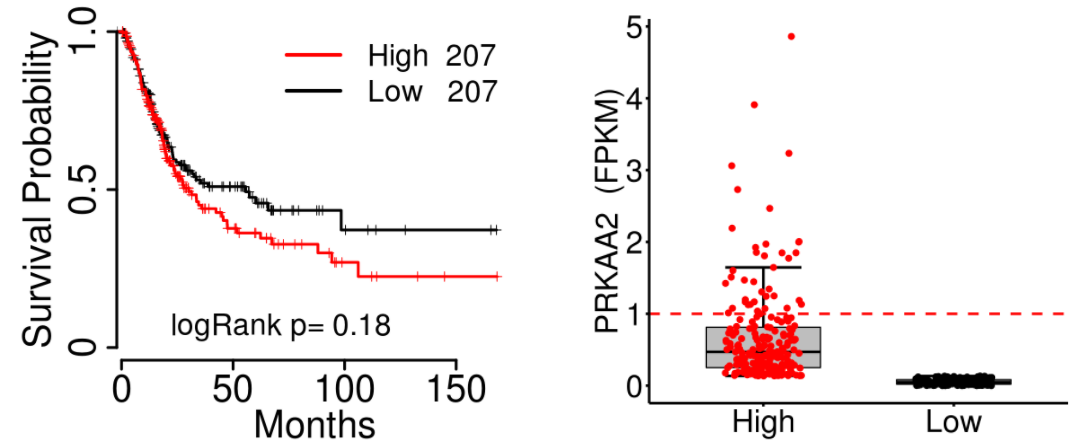


**Figure S3.** The survival probability and expression of Protein Kinase AMP-Activated Catalytic Subunit Alpha 2 (PRKAA2)
